# Dental Education

**Published:** 1903-04-15

**Authors:** C. N. Johnson

**Affiliations:** Chicago, Ills.


### DENTAL EDUCATION.

BY C. N. JOHNSON, L.D.S., D.D.S., CHICAGO, ILLS.

Read before the Chicago Dental Society, November, 1902.

The subject of dental education has for the past decade been claiming more than ordinary attention from the profession, and its interest seems to be constantly increasing instead of abating. It is the one supreme question which holds the minds of men in an ever-advancing ratio while other questions have their brief day of discussion and then wane away and are apparently forgotten. The reasons for this are that systems of education in general are being more assiduously studied in these latter days than ever before, and that dental education in itself is really one of the most significant factors connected with the advancement of our profession. In the present paper the author attempts not so much to formulate any radical theories of his own as to advance some ideas gathered from his intercourse with others and possible to flavor those ideas with impressions gained in his experience as a teacher.

Probably the question of all others which has aroused the greatest discussion is the one of preliminary training, not only with reference to the standard that shall be demanded of students for matriculation, but as to the precise nature of the studies which shall be taken up preparatory to entering a dental college. This question, with all the study that has been given it, may yet be considered in a chaotic stage, and it is one which should command the studious attention of every man who has at heart the welfare of the profession. It is fundamental in character, and strikes at the very root of our entire system of dental education. The first comprehensive thought which impresses one in this connection is that our present age is one of intense utility. Unless a thing can be made useful it is not wanted, and the lofty old adage "culture for culture's sake" holds small sway in the onward sweep of our apparently irresistible practicality. Much as the literary elect may lament the passing of this sublime sentiment the world will have little of it to-day, and we are forced to face a condition instead of fondly dreaming over a theory. And possibly in its ultimate analysis this is not so deplorable a thing after all, for what is culture in the best sense but the highest development of the intellect toward some utilitarian attainment?

If then in the preliminary training of students of dentistry the aim should be in the direction of the greatest utility the question arises as to what constitutes the most useful studies for a dental student to take up. To go back far enough it may be said that the instruction given in our common and high schools is along the right lines so far as it goes. Starting at the average age of six, a pupil should in the ordinary course of events, be able to graduate from a high school at from seventeen to nineteen, and no student should be accepted in a dental college short of this requirement. He will then have obtained at least a fundamental knowledge of such subjects as history—ancient, medieval and modern—grammer, literature, rhetoric, the languages, mathematics and elementary physiology and chemistry. There is not a single subject in this list which should not enter into the preliminary training of a dental student, for no matter how much such an individual may wish to specialize later on in his work the basis of his education should be broad and comprehensive. If we are to attain to any distinction in the professional world we must have men mentally equipped to assume professional status. But there is one thing significant of dentistry apart from other professional pursuits, and that is that our specialization should begin earlier in life than with the others. The character of our work is such that there must be developed in the individual the very highest degree of manual dexterity, and to attain this in the greatest profession the fingers should be trained in youth when the muscles are in their formative stage and can readily be directed into the proper manipulative procedures.

As I view the subject of preliminary training for dental students there are two dangers constantly confronting us either that our students shall lack a sufficient mental development along purley educational lines to entitle them to respect as professional men, or that in the attempt to remedy this the purely mental education shall be carried along without the concomitant manual development to an age where this manual development is exceedingly difficult or well-nigh impossible of attainment. As a result of this dilemma we have to-day in our ranks men who are skillful manipulators of instruments, but who are so deficient in intellectual acumen as to be really a reflection on the profession, and we have other men who though well trained mentally lack the adequate manipulative ability to enable them to properly perform the many difficult and intricate mechanical operations necessary for the highest class of service to their patrons. As to which of these two classes it is the more desirable to have in the profession the present paper has nothing to do, but the fact, remaining as it does, points to a peculiar difficulty in the training of students for the future practice of dentistry. Much good can be accomplished in this direction by a judicious arrangement of the pupil's studies in the later years of his high school work, and this duty devolves for the most part on the members of the dental profession. It is usually the case that a boy begins to think something about the profession he wishes to follow when he is fifteen or sixteen years of age, and it is the natural thing for him to consult with members of that profession as soon as he decides that he wishes to take it up. If a boy thinks he wants to be a dentist he goes to the family dentist to talk it over, and it is just at this point that the members of the profession can do great good if they take an active interest in the matter and inform themselves as to the proper preliminary lines to suggest for a student to follow.

With the consensus of opinion among dental teachers as a basis to guide the practitioner in the advice he shall give under the circumstances, the work of the young man will be directed in such a manner that when the time comes for him to enter the dental college he will be so equipped that he can intelligently approach his special work with the least possible waste of energy or misdirected effort. It may not be out of place at this time to very briefly suggest a few of the things which should be emphasized in the preliminary training of such a student. He should first be impressed with the fact that above all things else the fundamentals of his education should be broad, that if he is to be a professional man in the truest sense he must have a cultivated mind. He should therefore study literature for his general culture, and if he does nothing else in this connection he should at least learn to use his native language without offense to its grammar or construction. A professional man who constantly sins against the simplest proprieties of ordinary speech is not well calculated to impress the public with the dignity of his calling In line with the specialization which should begin in his preparatory work there are two subjects which stand out as distinct necessities—the study of physics, and a course in manual training. The laws of force, matter in motion, resistance to stress and kindred topics, all have a direct bearing on his future work, and as for manual training it is almost universally acknowledged to be the one essential equipment for the successful study of dentistry. It is true that some observers claim that this training may be given—as it is for the most part now—after the student has entered the dental college, but the danger lies in the fact that if deferred too long manual dexterity is exceedingly difficult of attainment. While it may be a fact that in some instances we see men beginning the study of dentistry at a comparatively mature age and yet making skillful operators it will invariably be found that such men were either previously engaged in pursuits which gave them manual training or else they were by nature mechanically inclined. There is one thing certain that no dental student either young or old ever gets too much development along manipulative lines, and it is quite generally conceded that the younger this development begins the more certain are the results attained. That this principle is clearly recognized by teachers in other fields is well illustrated by the remark of a prominent teacher of music recently to the mother of a boy of fourteen who was about to start the study of the piano. Said the teacher: "Your boy has lost at least seven years in finger development."

It is along these lines then that we should have our students trained so far as specialization is concerned before their entrance to a dental college, and it is confidently believed that if students were generally so directed they would come to us much better equipped than they do to-day.

Another important consideration connected with dental education relates to the order of studies that shall be taken up in the different years, but as this is a subject more suited to a teaching body than to a regular society it must be passed here with the simple statement that unless our preliminary training is revised in accordance with advanced ideas, a very radical change is needed in the arrangement of the college curriculum. As it is now the freshman year is crowded too full of abstruse and difficult subjects, to the utter confusion and disheartenment of many of our students. They are too suddenly plunged into the intricacies of such subjects as physiology, anatomy and chemistry, and are at this stage of their study utterly unable to trace any relationship between these subjects and their future life work. The grading of our college studies should be changed unless students come to us better equipped in mental training than most of them are. This subject is strongly commended to the careful consideration of such bodies as the National Association of Dental Faculties, and the Institute of Dental Pedagogics, with the hope that when the four year course is inaugurated a more equitable and utilitarian arrangement of the work will be brought about.

There is another reform needed in connection with the curriculum of our dental colleges, and which if we study the matter from the broadest point of view will be found to apply equally well to nearly all lines of professional education. It relates to the admission of all classes of applicants to the same grade irrespective of their previous work, provided, of course, that the minimum requirement for matriculation is presented. In other words a young man may go to Harvard, Yale, Princeton, Cornell or the Chicago University and graduate. During his university course he takes up the study of chemistry, for instances, and pursues this study for one or two years of his course. This young man enterrs a dental college and he is placed in precisely the same grade as a man who has not even the merest smattering of chemistry, and whose only recommendation is that he barely squeezed through his matriculation requirements. This is not right, and my temerity in saying so is justified by the fact that other professional schools err in the same way. It is a fundamental fact of equity and justice that a young man should be given credit for the knowledge he possesses irrespective of where he obtained it, and it is probable this one discrepancy in our requirements which has heretofore kept university graduates in many instances from entering dental colleges. There must of course be placed about a candidate for admission such restrictive measures that it will be impossible for him to assume advanced standing beyond his legitimate status, but when this status is once sufficiently proved he should be placed where he belongs, and not be obliged to go back to the beginning alongside of men who have as yet no status.

In line with this idea there should also be a reform in the grading of our students after they have entered college. It is a well known fact that there is the very greatest difference among students as to their native ability in the acquirement of knowledge and technical skill, that one student will readily grasp everything that is taught to him while another requires iteration and reiteration in a compound ratio in order to force anything into his intellect. It is not right to hold the bright student back on account of the limitations of the dull student by his side, and while this seems to be to a certain degree unavoidable in any kind of instruction where class work is followed, it should be the aim of dental colleges to remedy this as much as possible by making the element of qualification rather than that of time the basis of requirement for advanced standing or graduation. It would probably at this time be too radical a change to suggest that the time element be ignored, and to say that when the student has properly fitted himself for practice he should be given his diploma irrespective of the time spent in acquiring his knowledge. This would to some extent be a retrograde movement unless every college could be depended upon to discriminate carefully in its examinations and hold rigidly to the highest possible standard of attainment as a basis for graduation. But one thing the colleges can and should do even under the present system of a minimum three or four years course, and that is to hold frequent examinations for advanced standing throughout the course and grade their students rigidly according to this test. It is folly to admit a student to advanced standing year after year when his only title thereto is the barely eked out number of marks for a pass, probably given him as the benefit of a doubt on the part of his examiner and this frequently with a downright condition on one or two subjects for him to make up. Such a student is handicapped through his entire course and is never capable of doing his best work. It is far better that he be placed back at the outset and made to do over again the work in which he is found deficient. If this were done the students would soon learn that it was a question of qualification rather than one of time, and would accordingly work with a more intelligent relationship to their responsibility in the matter.

And this brings up another subject which has been made the basis of much discussion in the past, and which has not by any means been settled yet. This relates to the advisability of culling out the undesirable timber among our students in the first year of college work or better yet, before college work begins. It would, of course, be a beneficent provision if it were possible to prevent a student whose natural endowments or lack of endowments were such that he could never succeed as a dentist, from wasting his time in the attempt to study the profession. But the question arises as to who is to judge of so intricate a problem as this? I must confess as a teacher of some experience and observation that I am not so sanguine of my ability in this particular as I formerly was. It seemed to me in the early years of my teaching that I could judge quite accurately of a student's ability to become a capable dentist by watching him in the first few months of his college course, but I am no longer so certain of it. I have had my judgment so frequently set at naught by these unpromising students, that I should hesitate long and guardedly before condemning one of them to banishment from our college walls.

The simple fact is that the requisites for the successful practice of dentistry, though quite numerous and very exacting, are yet for the most part of a nature which can be cultivated, and while this at first thought may not seem to be true, a careful analysis of the question if we had the time to consider it here would prove it to be the case. Granted, then, that this is a fact there is one characteristic of a student which counts for more than anything else in his ultimate attainment, and that is the ability and inclination to plod. The sublime ingredient in a human make-up which impels the individual to constant application will do more to make a good dentist than any amount of native ability devoid of this characteristic. And so if I were called upon to judge a student as to his adaptability for the study of dentistry I should look to the question of his attitude toward his work—whether or not he was in earnest and really wanted to be a dentist, and whether he had unlimited patience and perseverance, rather than to the single question of his manifest mental and manual qualifications. This does not imply that there are not many men entering our ranks who would better be kept out, but merely that the selection of the proper material and the rejection of the culls is not so simple a matter as many in the profession have considered it. —*Review*.

